# The Victims of the War

**Published:** 1902-06-07

**Authors:** 


### The Hospital

A Journal of

### The Medical Sciences and Hospital Administration.

Vol. XXXII.—No. 819. Edited by Sir Henry Burdett, K.C.B., and Solomon C. Smith, M.D., M.R.C.P. [June 7, 1902.

### The Victims of the War.

The termination of the war has given rise to exuberant expressions of joy all over the country—not so much of triumph or exultation as of gladness that the war is over. Perhaps to some extent this is due to the character of the struggle and to the nature of the reports sent home by many of the newspaper correspondents. The exigencies of the censorship in regard to matters military have driven some of the correspondents to turn their pens towards hospitals and such-like gruesome details, and one can hardly doubt that the prominence given to medical affairs in all the reports from the Front has done much to drive into people at home the distressing rather than the exciting side of war. Not only has the list of our losses been a long one, but these losses have told of fevers and weary illnesses patiently nursed and patiently endured, the unexciting miseries of disease rather than of heroic struggles and deeds of daring on the field of battle. Taking deaths alone, while 5,776 officers and men are put down as killed in action, and 2,019 as having died of wounds, no fewer than 13,272 are stated to have died of disease. We have indeed heard so much about fevers and the like that some people have run away with the idea that microbes rather than bullets have constituted the chief danger in this war. When compared with other wars, however, this is found not to have been the case. Take, for example, the conflict between Russia and Turkey in the years 1877 and 1878, the last great European war. This was a war of big battles, the frightful slaughter before Plevna being an example of all that is most horrible in warfare. Yet in the Russo-Turkish war, which lasted only 18 months, and in which 505,000 Russian troops were engaged, the Russians lost roughly 88,000 from disease against 28,000 from wounds. Thus, while our deaths from disease have not quite doubled our deaths from wounds, more than three times as many Russians died of disease as from wounds during their much shorter war, and this notwithstanding the slaughter which took place time after time under the guns of Plevna and in the Balkans. It is, however, when we look at the ratio between the losses by disease and the total number engaged that we see how comparatively good has been the health of our troops in South Africa, the deaths from disease among the Russians in 1897–98 having been more than four times as numerous, in proportion to the numbers engaged, as they have been among our troops during the recent war. When we turn from the question of disease to that of wounds one thing comes out in a very striking manner—namely, the very high ratio of officers to men who were killed in action or died of wounds, and the high ratio of men to officers among those who died from disease. Among those killed in action there seems to have been one officer to 10·15 men, while among those who died of wounds there was one officer to 11·34 men; but among those who died from disease there was only one officer to 38·5 men—a very different ratio. In other words, among the sick there was perhaps a somewhat larger proportion of men, while among the killed and mortally wounded there was a far larger proportion of officers than obtains in the Army generally.

Many causes have conspired to produce this excessive loss of officers on the field. It is evident that leaders must always be more exposed than men, and in the early stages of the war the officers were obviously made targets of by the enemy. There seems but little room for doubt, however, that many young lives were thrown away in the mad race for the Victoria Cross, a decoration which, although instituted as a reward for valour, has too often acted as an incentive to reckless daring. All honour to the brave lads! but one is sorry. Finally let us not forget the long list of invalids who have been sent home, somewhere about 70,000 in all, more than 6,000 of whom have since died or left the service, while probably all have had their lives shortened and their usefulness diminished by the sufferings they have undergone. They also are victims of the war. The vastness of the medical work and the immensity of the suffering which the medical department has struggled to relieve in the course of the war are difficult to realise. And yet, be it remembered, as wars go, this has not been a great one. If we want to know what war really means we must think of Gravelotte, where in one single battle the victorious Germans lost 328 officers and 4,900 men dead on the field, besides nearly 15,000 wounded, not so very far from the total of those killed in action during the whole of this South African campaign. Let us thank God that we are now at peace, and let us pray that we may never be involved in what our friends upon the Continent would call a real war.

